# How to clean a catheter: Development of an intervention for intermittent catheter reuse

**DOI:** 10.1002/bco2.487

**Published:** 2025-02-04

**Authors:** Sandra Wilks, Margaret Macaulay, Jacqui Prieto, Miriam Avery, Catherine Bryant, Debbie Delgado, Cathy Murphy, Nicola Morris, Mandy Fader

**Affiliations:** ^1^ School of Biological Sciences University of Southampton Southampton UK; ^2^ School of Health Sciences University of Southampton Southampton UK; ^3^ Bristol Urological Institute North Bristol NHS Trust Bristol UK

**Keywords:** intermittent catheters, medical devices, reusable, single use, sustainability

## Abstract

**Background and Objectives:**

Much intermittent catheterisation (IC) is carried out using single‐use catheters. Waste and costs could be reduced by cleaning and reusing catheters, but is it safe to do so? To answer these questions of safety and sustainability, clinical trials are needed. In this study, we developed a user‐tested catheter cleaning method and training materials for use in a clinical trial.

**Methods:**

Focus groups selected candidate cleaning methods and developed draft instructions. Users then home tested these methods on uncoated, plastic‐based catheters, which were cleaned and reused up to 28 times. Reused and cleaned catheters were analysed using advanced microbiological analysis methods. The refined cleaning method was further tested by a naïve user panel. Additionally, a silicone catheter designed for reuse was tested in the laboratory and for user acceptability. User panel feedback was gathered throughout testing and thematically analysed.

**Results:**

Twenty‐six IC users were recruited to three user panels. Focus groups identified soap and water (SW) and soap and water plus a 15‐minute soak in a chlorine‐based cleaning solution (SW‐Cl) as the preferred cleaning methods. User testing (≤3 reuses) and laboratory analysis showed SW alone to be less effective than SW‐Cl: bacteria were detected in 23/120 (19%) male and 56/108 (52%) female SW samples versus 16/228 (7%) and 16/201 (8%) for SW‐Cl. Bacteria were detected in only 1/240 (<0.4%) of catheter samples after 8–≥28 reuses with the SW‐Cl method. Naïve user panel results were similar. The silicone catheter was acceptable to users and had comparable laboratory results using SW‐Cl. User panel feedback informed refinement and simplification of the SW‐Cl cleaning method and instructions.

**Conclusion:**

A chlorine‐based method for cleaning catheters, which effectively removed bacteria from catheters reused multiple times, has been developed, tested and refined by users, and captured in an instruction booklet and video for inclusion in a clinical trial.

## 1 INTRODUCTION

Incomplete emptying of the bladder is a common urological problem.^1^ If there is no treatable cause, the standard method of management is to teach the individual or their carer, clean intermittent catheterisation (IC). IC is the term used to describe the passing and removal of a urinary catheter into the bladder to drain urine. The process is repeated as needed according to urine output and bladder capacity, typically 4–6 times per day.^2^ IC has transformed the lives of many people with bladder emptying problems and is accepted as the optimum strategy when corrective treatment is not possible.^3^

IC was first described in the literature using catheters cleaned and reused multiple times by an individual user.^4^ Worldwide, there is variation in practice and while IC using single‐use catheters is standard in many countries, in others (e.g. Australia, Canada and the United States),^5^ some reuse of catheters continues. In 1993, the EU Directive on medical devices^6^ required reprocessing (cleaning) instructions for products intended for multiple use. Intermittent catheters prescribed in the UK were subsequently supplied sterile with labelling indicating single use.

In recent years, the increase in concern about the use of single‐use, plastic‐based products has brought into question the impact of single‐use catheters.^7^ The environmental cost of using single‐use catheters has been estimated to be around 206 million litres of waste per annum in the United States.^8^ Analysis of NHS prescription data shows that, in England alone, around 100 million catheters are thrown away each year.^9^ Additionally, the cost of single‐use catheters has risen as the number of IC users has grown. In England, the prescription costs of single‐use catheters increased by 70% from £97 m in 2014 to £166 m in 2022.^9^ Because they can be used multiple times and are not discarded after use, reusable catheters may be cost‐effective^10^ but this is not certain. A recent Cochrane review reports that no included trials of reusable catheters have undertaken health economic analyses.^11^ To determine cost‐effectiveness, the health‐care costs from infections, hospitalisations, trauma and cleaning costs would need to be evaluated, as well as the costs of the catheters.

Interviews with IC users (who mainly used single‐use catheters)^12^ revealed that although there are advantages to single‐use catheters (e.g. instantly useable and convenient), there are also some disadvantages (lots of catheters to carry around and throw away). Conversely, there could be potential advantages to reusable catheters (fewer to carry around, not running out), but an important potential disadvantage is that they may lead to more urinary tract infections (UTI). A Cochrane review found that there was insufficient evidence to determine whether a single‐use or reusable catheter strategy was better for UTI.^11^ Avery et al.^12^ concluded that mixed use, that is use by an individual of both single‐use and reusable catheters, could optimise the perceived advantages and disadvantages of both strategies, but further evidence was needed from users who had tested both catheter types.

Research into reuse is hampered by the lack of intermittent catheters designed specifically for reuse and clinical uncertainty about recommending them.^13^ Plain uncoated catheters for single use continue to be reused in some countries, but very few purpose‐made, reusable catheters exist. Most single‐use catheters are supplied with some form of integral lubrication or coating, which means that they cannot be reused. Reusable catheters are not supplied with lubricants or coatings, which must be added separately if required.

A further research challenge is the lack of published, rigorously tested methods for cleaning; these would be required to test the safety and effectiveness of using reusable catheters. Wilks et al.^14^ used multiple laboratory tests to determine the effectiveness of eight potential catheter cleaning methods identified in the literature, at removing bacteria from catheters. The next step is to test which of the most effective methods are both acceptable to users and effective at removing bacteria in practice.

## 2 AIM

The aim of the present study was to develop a user‐tested and effective catheter cleaning method, with training materials, to be used in a subsequent clinical trial of reusable catheters.^15^

Our objectives were to:

1. Identify one or more preferred methods, components, and products for cleaning, storing and lubricating catheters, and draft preliminary instructions.
2. Identify and implement any modifications needed to improve the utility and acceptability of the cleaning method(s).
3. Determine the effectiveness of the cleaning method(s) over increasing numbers of catheter reuses.
4. Test the effectiveness and acceptability of the final cleaning method used with plastic‐based catheters.
5. Test the effectiveness and acceptability of the final cleaning method on a silicone catheter licensed for reuse.
6. Create training materials including booklets and a video to be used in the trial.

## 3 METHOD

**FIGURE 1:**
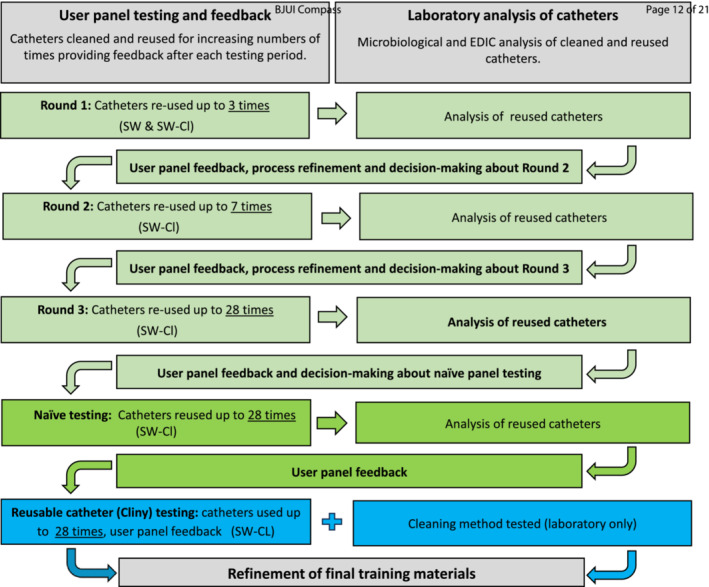
Methods and process for participant testing and feedback, and laboratory analysis (EDIC = episcopic differential interference contrast; SW = soap and water; SW‐Cl = soap and water plus a 15‐min soak in a chlorine‐based cleaning solution).

In line with the guidance on the development of complex interventions,^16^ we used a systematic and iterative approach. We worked with groups of IC users (three user panels) combining user testing and feedback, and laboratory testing, to identify and develop the best (most effective and most acceptable) cleaning method. User panel testing comprised repeated rounds of home testing with feedback via focus groups in preparation for and after each testing round. Figure 1 shows the methods of rounds of user panel testing and feedback.

## 4 PARTICIPANT RECRUITMENT

### 4.1 Participant identification

Potential participants (up to 10 per panel) were identified from urology outpatient clinics and community continence clinics within the Bristol and Southampton regions of the UK, and a national UK consumer organisation (Bladder & Bowel Foundation). Patients were sent a written invitation from the Chief Investigator or responded directly to the research team following contact from the consumer organisation. Eligible patients were sent a participant information sheet and provided written consent.

### 4.2 Eligibility criteria

*Inclusion criteria*: Over 18 years, currently using IC daily for more than 3 months, able to clean catheters and return them to the research team using pre‐paid mailboxes, able to provide written consent, no UTI at the time of testing.

*Exclusion criteria*: Urethral stricture, immune deficiency disorder, IC performed by an external carer/community nurse requiring the use of a sterile technique and catheter.

### 4.3 Ethics

NHS ethics approval was given by South Central – Hampshire A Research Ethics Committee REC reference 19/SC/0334.

## 5 USER PANEL TESTING, FEEDBACK AND LABORATORY ANALYSIS

### 5.1 Identification of one or more preferred cleaning methods, and the components and products for cleaning, lubricating and storing catheters (Objective 1)

We used a series of focus groups to show and discuss with a user panel (user panel 1) the potential cleaning methods previously identified and tested in the laboratory by Wilks et al.^14^ (boiling,^17^ microwave,^18^ steam, soap and water wash plus a chlorine‐based cleaning solution,^19^ vinegar,^20^ ultrasonic, soap and water,^18^ and tap water rinse) and to select their preferred methods. These focus groups were also used to show and discuss (a) a range of plain, uncoated, single‐use, plastic‐based intermittent catheters (catheter) that could be reused; (b) a range of lubricants; and (c) potential component parts and equipment (kit) needed for the cleaning method. They chose their preferred catheter and lubricant from the selected range. They also reviewed and refined preliminary draft instruction materials. Using the preferred cleaning methods, preliminary kit, rounds of user and laboratory‐based catheter testing followed.

### 5.2 User panel testing process (Objective 2)

The most effective^14^ and preferred cleaning methods were used by participants to clean and reuse catheters for an increasing number of reuse events (maximum 28 times) over a series of testing rounds (Rounds 1–3).

The participants were given all items necessary for using and cleaning the catheters including refined preliminary instruction materials. After use, the participants returned their cleaned catheters to the laboratory in Royal Mail safe boxes for microbiological analysis. They collected urine samples using their own sterile catheter at the start of testing and at the start of each round. These samples were returned to the laboratory as above (see Supporting Information 1).

The number of reuse events was increased between rounds until a catheter had been used up to 28 times (*Round 1*: up to 3 times; *Round 2*: up to 7 times; *Round 3*: up to 28 times). Progression to the next round was determined by (a) <10% catheter samples having detectable bacteria and (b) positive participant feedback.

### 5.3 User panel feedback (Objective 2)

Qualitative data were obtained from user panel 1 via single sex focus groups who met before and between testing rounds. Feedback was sought about all aspects of the cleaning method and associated processes necessary for catheter reuse including catheter lubrication and storage. Data were recorded electronically or by video, or by detailed note taking. Audiotapes were transcribed verbatim. Data were sorted into themes and sub‐themes and used to develop and inform modifications to the cleaning method and guidance.^21^

### 5.4 Laboratory analysis (Objective 3)

At the laboratory, returned catheters from all testing rounds were first inspected for any visual signs of damage, discolouration or the presence of deposits. Catheters were divided into three (tip, mid and base) sections, and those sections and urine samples were analysed using advanced microbiological methods (see Supporting Information 2). Culture analysis was carried out in triplicate on all catheter and urine samples.

Episcopic Differential Interference Contrast (EDIC) microscopy^22^ was used to examine the external surface and internal lumen of each returned catheter (see Supporting Information 3).

### 5.5 Naïve user panel testing (Objective 4)

The participants not previously involved in testing (user panel 2), reused catheters up to 28 times using the most effective cleaning method from previous stages of testing to gain a fresh perspective and critique of the cleaning method.

### 5.6 Reusable catheter (Cliny) user panel testing (Objective 5)

Regulatory issues subsequently prevented single‐use catheters being prescribed off‐licence for reuse in the trial. Therefore, the silicone Cliny catheter (Create Medic Co., Ltd.), CE‐marked and licensed for reuse up to 28 days, was tested under laboratory conditions (see Supporting Information 4). It was also tested for acceptability by a small user panel (user panel 3) who reported their experiences.

### 5.7 Refinement of final kit and training materials (Objective 6)

The final kit and written materials were reviewed by two men and two women who had tried out the Cliny catheters. These were refined and the manufacturer's instructions for using the Cliny catheter were incorporated. The final instruction booklets were prepared for use in the MultICath trial.

## 6 RESULTS

### 6.1 Participants

Fifteen men (mean age: 68 years; range 51–88 years) and 11 women (mean age: 58; range: 38–70 years) were recruited. During testing, four men and one woman withdrew for non‐catheter related reasons and one because they found the reusable catheter was too uncomfortable to use.

### 6.2 User panel testing, feedback and laboratory analysis

#### 6.2.1 Identification of preferred cleaning methods and development of the kit for cleaning, lubricating and storing catheters (Objective 1)

We held three focus groups each comprising a total of five men and four women (user panel 1).

The user panel rejected six cleaning methods which they considered to be impractical requiring heat or kitchen facilities and/or laboratory testing had shown to be ineffective (boiling, microwave, steam, vinegar, ultrasonic and tap water rinse). Two methods were selected by participants for testing. Soap and water (SW) and soap and water plus a 15‐min soak in a chlorine‐based cleaning solution (SW‐Cl) methods were selected (see Supporting Information 5). The SW‐Cl method was considered by participants likely to be the most practical cleaning method, but they also wanted to test out SW which was considered simpler.

Following discussions in the focus group, three plain uncoated catheters were selected for testing from the range available on the Drug Tariff (England & Wales prescribing formulary) (Huntercath, Hunter Urology now Optimum Medical®; Self Cath, Coloplast; Wycath, Wymedical now Flexicare). The three water‐based lubricants available on the Drug Tariff were selected for testing (KY jelly, Johnson & Johnson; Optilube, Optimum Medical®; Sutherland jelly, Sutherland®). The participants handled and commented on a range of potential products for catheter cleaning, drying and storage and agreed items for initial home testing—components are listed in Supporting Information 6. Developed and refined instructions for preliminary testing included men's and women's booklets and video, and a laminated ‘how to’ guide. User panel testing then followed (Figure 1).

#### 6.2.2 User panel feedback (Objective 2)

The participants provided detailed feedback about all aspects of the catheter cleaning and reuse method at focus groups held after each round of testing. These qualitative data were used to modify the items they were given for subsequent testing (see Supporting Information 7). For example, the participants expressed concern that the lumen of the catheter might not be cleaned by the SW‐Cl method. Items for cleaning the lumen were considered including a narrow, nylon micro brush, ‘catheter‐tip’ syringe and balloon syringe. The participants selected the balloon syringe for testing. Discrete ways of carrying products were also introduced; for men, this included a small, round container to enable catheters to be carried in pockets; for women, this included a pencil case for easy carrying in a bag. The participants found the chlorine‐based cleaning agent as a fluid inconvenient when away from home and the tablet version was provided as an option.

Analysis of the qualitative data gave useful insights into participants' experiences of reusing catheters (see Supporting Information 7). The participants acknowledged a burden from reuse but found that practice reduced this: *‘When I first started the trial, I did find it a little bit of a nuisance sterilising the catheters but towards the end it became so much easier it just became part of regular life really.’* Overall participants found there to be pros and cons to both single use and reuse of catheters: *‘Being able to use the option of using both the reusable and the single use just was peace of mind that I could use whichever one was most convenient depending where I was.’*

#### 6.2.3 Laboratory analysis (Objective 3)

##### Round 1

Laboratory analysis found the SW‐Cl method to be effective at disinfecting the catheters after up to three reuse events and more effective than SW alone (Table 1); 23/120 (19%) samples from male participants and 56/108 (52%) samples from female participants having culturable bacteria detected following SW only, compared to only 16/228 (7%) and 16/201 (8%) (male and female respectively) following the use of the SW‐Cl method. In all cases, the bacterial species found were the same as those detected in participants' urine. Participants reported the SW‐Cl method to be acceptable in practice. Therefore, the SW‐Cl method was adopted as the catheter cleaning method for subsequent testing.

**TABLE 1:** Microbiological analysis (plastic‐based catheters): number of samples from user panels 1 and 2 with culturable bacteria following cleaning with soap and water only (SW) or soap and water plus a 15‐min soak in a chlorine‐based cleaning solution (SW‐Cl), over increasing numbers of reuse events (for each catheter, three samples were analysed; the tip, the middle and the base).

No. times catheter reprocessed. (*N* participants)	Cleaning method used	Number of sections with culturable bacteria			
		Male (% samples with bacteria)		Female (% samples with bacteria)	
**1–3 (15)**	Soap and water only	23/120	**19.2%**	56/108	**51.9%**
	SW‐Cl method	16/228	**7.0%**	16/201	**8.0%**
**4–7 (10)**	SW‐Cl method	3/48	**6.3%**	0/174	**0%**
**8‐ ≥28 (9)**	SW‐Cl method	1/117	**0.9%**	0/123	**0%**

##### Round 2

Laboratory analysis continued to demonstrate the SW‐Cl method to be an effective disinfectant for catheters used for up to seven reuse events with 3/222 (1.35%) of catheter samples showing detectable culturable bacteria (Table 1). The participants continued to find the SW‐Cl method to be acceptable, although they had some concerns about the odour of the chlorine and possible skin contamination. They also felt that a luminal flush with chlorine would be important for thorough cleaning, and this was added to the cleaning method. Effective catheter drying was reported as difficult to achieve.

##### Round 3

Laboratory analysis revealed only 1/240 (0.41%) catheter samples to have detectable culturable bacteria after ≥28 reuse events (Table 1). The participants reported being able to manage catheter reuse well at home. They had varying degrees of difficulty re‐using catheters away from home, for example, transporting and lubricating uncoated catheters.

##### Urine analysis (Objective 3)

High levels of culturable bacteria were collected from the urine of all but one participant prior to reuse. All participants had experienced symptomatic UTIs previously but were not known to have any ongoing infections over the study period. For the participants at all stages, the most commonly found bacterial species was *Escherichia coli (E. coli)*, isolated from 57% of the samples followed by enterococci (31%), staphylococci (18%), streptococci (14%) and coliforms (14%). The identified bacteria tended to remain constant over the sampling times. One‐way analysis of variance (ANOVA) of the mean values across all samples found no significant differences in bacterial concentration regardless of the number of reuses (≥28).

##### EDIC analysis of reused catheters (Objective 3)

**FIGURE 2:**
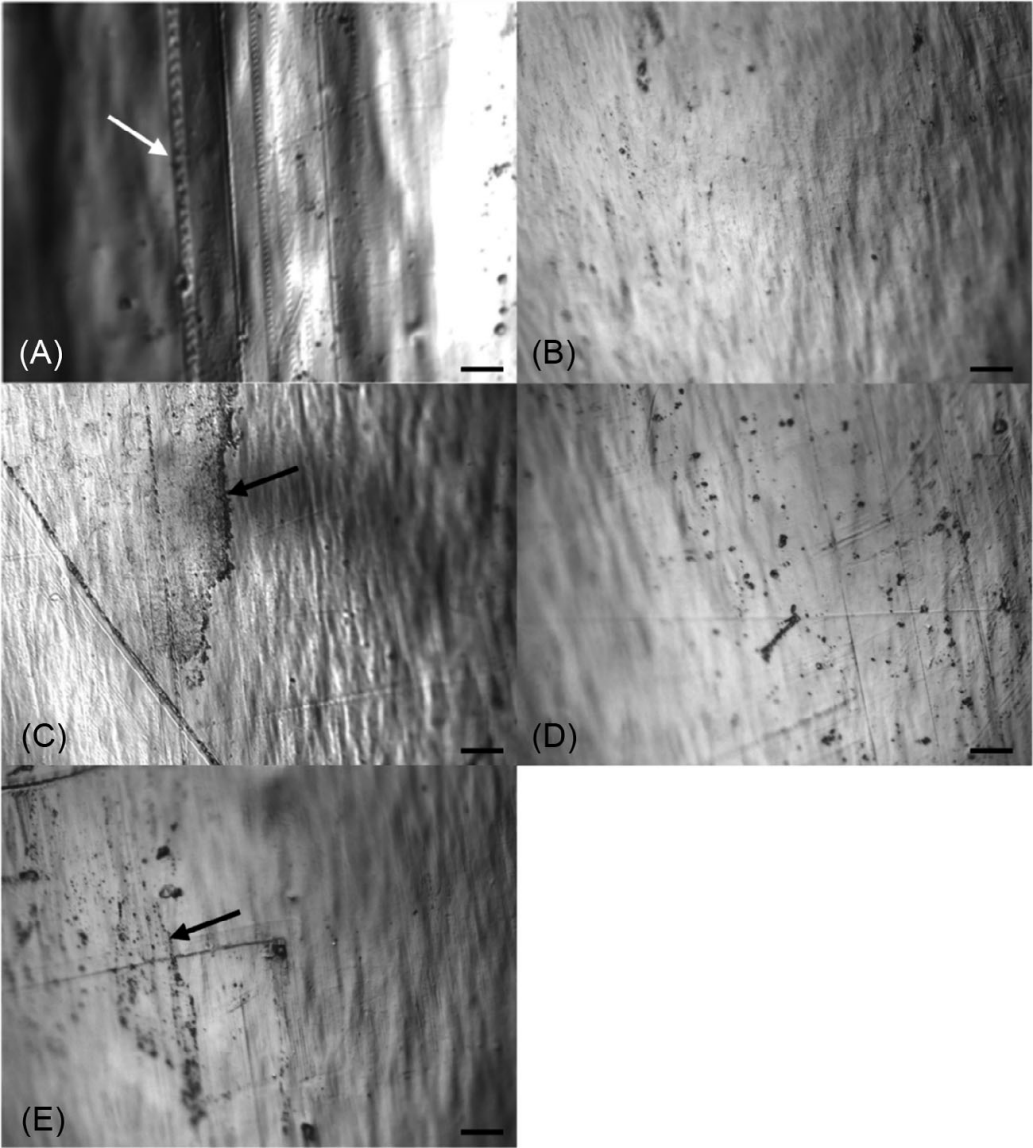
Episcopic differential interference contrast (EDIC) analysis. EDIC microscopy images of uncoated plastic‐based catheters before and after repeated use. All images show the external catheter surface. (A) Unused plastic‐based catheter, (B) following seven reuse and cleaning events, (C) following 14 reuse and cleaning events, (D) following 21 reuse and cleaning events, and (E) following 28 reuse and cleaning events. Cleaning with SW‐Cl method only. White arrow indicates scratches and inconsistencies found in catheter plastic‐based material before use, black arrows indicate areas with visible conditioning film composed of cellular debris and proteinaceous material. Magnification ×500, scale bar = 20 *μ*m.

No visible evidence of damage to the catheter surfaces or material was found on any catheter samples following repeated reuse and cleaning using the SW‐Cl method ≥28 times. The catheter sections did show some coverage with a conditioning film, where organic macromolecules and proteins coated the surface. This covered all catheter lumens, and some external surfaces, rapidly building up after only a few reuses (Figure 2) and continued to be present with increasing numbers of reuse events. The conditioning film was found to be denser closer to the tip and around the eyeholes. This indicates a clear relationship with the presence of urine, with evidence of host cells and microcrystalline deposits in some samples but no visible biofilm formation or presence of attached bacteria.

##### Naïve user panel testing (Objective 4)

Laboratory analysis showed the SW‐Cl method to be effective at disinfecting catheters cleaned and reused ≥28 times by the participants new to the procedure (user panel 2 – five men and four women) (Table 1). Novice participants found the method acceptable and reported nothing new.

#### 6.2.4 Reusable catheter (Cliny) user panel testing (Objective 5)

User panel 3 (two men and two women) tested the Cliny catheter for user acceptability over a 2‐week period cleaning it between uses with the SW‐Cl method. The participants found the Cliny catheter to be more flexible than plastic‐based equivalents, although most were able to insert them with practice: *‘People who have been trained to use more rigid catheters may find this very bendy catheter a bit tricky to get used to.’ (woman)* They were found to be acceptable for cleaning and reuse with the SW‐Cl method.

**FIGURE 3:**
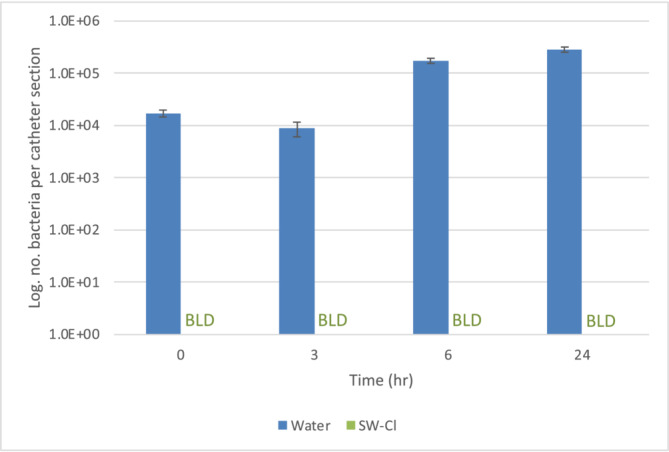
Microbiological analysis (silicone catheters). Number of culturable *E. coli* remaining after control water rinse or the SW‐Cl method with repeated exposure times. BLD (Below Limit of Detection) represents values below the limit of detection (67 bacteria per catheter section), recorded for all tests using the SW‐Cl method (for full method see Supporting Information 4).

Laboratory modelling analysis as in Wilks et al.^14^ found the SW‐Cl method disinfected silicone catheters as effectively as it did plastic‐based catheters (Figure 3). For all replicates and at all time points, culturable bacteria could be detected following a water rinse, increasing to approximately 10^6^ bacteria per catheter section after 6 h (representing three reuses). In contrast, at all time‐points, no culturable *E. coli* were detected following cleaning with the SW‐Cl method.

#### 6.2.5 Refinement of kit and training materials (Objective 6)

Modifications made to the catheter cleaning equipment resulted in a simpler ‘slimmed down’ kit comprising only three products for catheter cleaning (container, chlorine‐based cleaning product and syringe) and lubricant gel (Supporting Information 6). New versions of the booklets for men and women and a unisex video were developed for testing the silicone catheter. For the trial, final versions of the booklets for men and women were printed, and both these and the video made available on the MultICath Trial website.

## 7 DISCUSSION

This paper describes a novel iterative process. Following guidance on the development of complex interventions,^16^ we combined laboratory testing with qualitative methods (user panel testing and feedback) to develop a refined catheter cleaning method for the reuse of intermittent catheters. Other published studies have focussed on testing cleaning methods only under laboratory conditions.^14^ To our knowledge, this is the first study that has developed a cleaning method with users and tested the acceptability and effectiveness of the cleaning method in practice. Such a process could be used with other single‐use products, for example, leg bags.

Laboratory testing has demonstrated that a chlorine‐based cleaning method was effective at removing a range of bacterial strains from the surfaces of both plastic‐based and silicone catheters and reducing bacterial contamination to below a recordable threshold. Furthermore, repeated use and cleaning resulted in lower bacterial counts. This may have been because of participants becoming more skilful in the cleaning method and/or the cumulative effect of chlorine on catheter surfaces. In all cases, pre‐existing, high levels of culturable strains of bacteria found in the urine were the same as those found on the participant's catheters. This would indicate that it is the individual's own bacteria which contaminate the catheter. A conditioning film (composed of proteins and cellular debris) was found on the internal lumen of all used catheters, and on some external surfaces, but with no evidence of bacterial attachment. Development of the conditioning film is related to the presence of organic material in the urine, for example, cellular debris, which will attach to the catheter. In no cases did a biofilm develop. Furthermore, repeated cleaning with the SW‐Cl method did not damage the external or luminal catheter surfaces of different catheter types.

In the initial stages of the study, the participants expressed the need for many components of equipment that were thought to be important for the cleaning method. During testing, this number reduced as the participants desired simplicity and found that few components were essential. For some women, catheter lubrication with gel was unnecessary as they used water, the cleaning solution or nothing. The final version of the kit, excluding catheters, comprised five components for men and four for women not requiring a lubricant. All items are readily available for purchase from a chemist or, in the UK, via prescription from a GP. The reusable items (container and balloon syringe) cost in total approximately £13 (€16, $17) and could be expected to last for at least for one year. The cost of consumables (chlorine‐based cleaning product, soap and lubricant gel) would vary depending on the type and the amount of product used, and we estimate this to be approximately £5–10 per 28 days (€6–12 or $7–13). If a catheter cleaning kit were to be made commercially, refinements could improve the size, aesthetics and utility of the items, in particular to make it easier to use when away from home.

Reuse of the test catheters was not without its difficulties. These mainly related to using reusable catheters outside the home, and the need, particularly for men, for an external lubricant. These findings are in line with previously published perceptions of IC users about the potential advantages and disadvantages of reusable and single‐use catheters^12^ and the benefits of being able to use both types.

Reduction of waste and the environmental impact of health care is becoming increasingly important globally. The UK NHS has a target for net‐zero by 2045.^23^ Much of health care is carried out in the home, and methods that enable patients who wish to and can utilise more sustainable ways of managing long‐term conditions such as IC are needed. The participants in this research have demonstrated a willingness to develop catheter reuse options that could have the potential to reduce impact on the environment.

Repeated cleaning and reuse of catheters was once common but is now prohibited in the UK leaving IC users with single‐use options only. If catheter reuse was to become an option once again, it would be essential to have an evidence‐based, co‐developed catheter reuse intervention to give clinicians and IC users confidence in their use.^13^ The evidence‐based cleaning method developed in this research has been incorporated into the MultICath trial^12^ and is also available to industry to support the licensing of reusable catheters and to encourage catheter innovation.

### 7.1 Limitations

Because there are no catheters licensed for reuse in the UK, we initially used, with ethics and sponsor approval, single‐use, plain, uncoated catheters to develop the cleaning method. However, a clinical trial of reusable catheters requires a catheter licensed for reuse. We therefore tested a catheter that was CE marked for reuse only at the final stage.

Although we recruited the participants in urban and rural areas, there was limited ethnic and socio‐economic diversity within the group. This reflects a wider difficulty ensuring diversity in research generally.^24^ Differences such as physical disabilities and varying cultural norms may affect people's views about, and their ability to use, this cleaning method. The participants in this research can be assumed to be well disposed to catheter reuse, and thus the user views and feedback are likely to be more positive than might be the case for the wider intermittent catheter user population.

## 8 CONCLUSION

A catheter cleaning method to allow the multiple use of uncoated intermittent catheters has been developed with IC users and tested using advanced laboratory techniques. It has been demonstrated to be effective at cleaning plastic‐based and silicone intermittent catheters when used repeatedly by a small group of adult men and women. This method is suitable for testing in a large randomised controlled trial and has the potential to be used more widely if reuse is shown to be safe.

## AUTHOR CONTRIBUTIONS

**Sandra Wilks:** Microbiology (lead and work) and manuscript preparation. **Margaret Macaulay:** Study management, panel recruitment, data collection and analysis, and manuscript preparation. **Jacqui Prieto:** Infection control expertise and manuscript editing. **Miriam Avery:** Panel recruitment, data collection and analysis, and manuscript editing. **Catherine Bryant:** Microbiology work and manuscript editing. **Debbie Delgado:** Panel recruitment, data collection and manuscript editing. **Cathy Murphy:** Manuscript preparation and editing. **Nicola Morris:** Microbiology work and manuscript editing. **Mandy Fader:** Overall design, supervision and manuscript preparation and editing.

## CONFLICT OF INTEREST STATEMENT

None of the authors have a conflict of interest to disclose.

## Supporting information


**Supporting Information 1:** Microbiological analysis of urine
**Supporting Information 2:** Microbiological analysis of plastic‐based catheters
**Supporting Information 3:** Episcopic differential interference contrast (EDIC) analysis
**Supporting Information 4:** Microbiological analysis of silicone catheters
**Supporting Information 5:** Test method for cleaning catheters using soap and water and chlorine‐based cleaning solution (SW‐Cl)
**Supporting Information 6:** Components reviewed for catheter cleaning and associated procedures necessary for catheter reuse.

**Supporting Information 7:** Summary of user feedback, example quotes and modification to cleaning method and other processes for catheter reuse (separate file)
