# Supplementary material for: How to clean a catheter: Development of an intervention for intermittent catheter reuse
Source: BJUI Compass. 2025 Feb 4;6(2):e487. doi: 10.1002/bco2.487 (PMC11794242; doi:10.1002/bco2.487)

**SUPPLEMENTARY MATERIAL**

Supplementary material 1: Microbiological analysis of urine

Supplementary material 2: Microbiological analysis of plastic-based catheters

Supplementary material 3: Episcopic differential interference contrast (EDIC) analysis

Supplementary material 4: Microbiological analysis of silicone catheters

Supplementary material 5: Test method for cleaning catheters using soap and water and chlorine-based cleaning solution (SW-Cl)

Supplementary material 6: Components reviewed for catheter cleaning and associated procedures necessary for catheter reuse.

Supplementary material 7: Summary of user feedback, example quotes and modification to cleaning method and other processes for catheter reuse (separate file)

1. **Microbiological analysis of urine**

Urine samples were collected at the start of testing and at the start of incremental increases in reuse events. The samples were serially diluted in 1/4 strength Ringer's solution and plated on to tryptone soya agar (TSA, Oxoid, UK) to quantify the total bacterial population, and Brilliance Clarity UTI agar (Oxoid, UK) to identify key bacterial uropathogens. Agar plates were incubated at 37°C for a minimum of 16 hours before colonies were counted to give colony forming units (cfu) per ml urine.

1. **Microbiological analysis of plastic-based catheters**

Cleaned, used catheters were returned to the laboratory.  Catheters were first inspected for any visual signs of damage, discolouration, or the presence of deposits. Three sections were cut from just below the tip (under the eyeholes), in the centre and adjacent to the base. For rounds 1-3, these sections were 2 cm in length and kept intact. Each of these sections was placed in 10 ml 1/4 strength Ringer's solution. For the Cliny testing, 1 cm sections were taken and cut longitudinally. One cut section was placed into 10 ml 1/4 strength Ringer's solution. The other cut section was kept for direct microscopy using an EDIC microscope. Attached bacteria were removed from sections in Ringer's solution by sonication or vortexing with glass beads. All samples were plated on to TSA and Brilliance Clarity UTI agar and incubated at 37°C. Visible colonies were quantified after 16 - 24 h incubation.

1. **Episcopic differential interference contrast (EDIC) analysis**

Episcopic differential interference contrast (EDIC) microscopy is a non-destructive advanced microscopy technique which allows direct examination of curved, opaque surfaces at high magnification (21). Catheter sections were examined, with no additional sample preparation, using an EDIC Nikon Eclipse LV100VD microscope (Best Scientific Ltd, UK) with metal halide light source (EXFO X-CITE 120), long working distance metallurgical objectives and a the QImaging Retiga EXi Cooled Digital CCD camera. Sections were scanned at low magnification and representative images taken at high magnifications of x500 and x1000 (total magnification). This method shows topographical structure of the catheter material, development of any conditioning form, and whether bacterial attachment and subsequent biofilm formation has occurred.

1. **Microbiological analysis of silicone catheters**

The Cliny all silicone catheter was tested under laboratory conditions using the method described by Wilks et al. (14). In summary, small sections of the catheter were exposed to a high concentration of uropathogenic *Escherichia coli* in an artificial urine medium for 5 minutes. These were then cleaned using either a water rinse (control) or chlorine-based cleaning solution. Following this time, sections were removed and either set aside for analysis or stored for subsequent re-exposure. This process was repeated at times 3, 6, and 24 hours. For analysis, one section was examined using episcopic differential interference contrast microscopy and the other had any attached bacteria removed and samples plated onto culture media to give colony forming units per catheter section. Three replicate experiments were completed.

1. **Test method for cleaning catheters using soap & water (SW) and soap and water plus a chlorine-based cleaning solution (SW-Cl)**

For the soap and water only wash (SW), catheters were cleaned between uses by washing with soap (standard commercially available liquid soap was provided but any soap was allowed) and tap water using hands lathered in soap or in a hand basin containing soap and water. Catheters were then rinsed with tap water and allowed to dry. For the SW-Cl method, we used a chlorine-based cleaning solution (Milton fluid or tablets) diluted according to manufacturer’s instructions with tap water resulting in 120ppm free chlorine. We followed the [Milton cold-water method](https://www.milton-tm.com/en/consumer/sterilising-methods): catheters were washed with soap and water as above and then immersed in the Milton chlorine-based cleaning solution. This solution was also flushed through the lumen using a plastic Pasteur pipette. Catheters were left to soak for a minimum of 15 minutes and were rinsed with tap water after treatment.

**COMPONENTS REVIEWED FOR CATHETER CLEANING AND ASSOCIATED PROCEDURES NECESSARY FOR CATHETER REUSE.**

**Total number of components at commencement of user testing = 14 items (excluding catheters)**

**Cleaning**

- Chlorine-based cleaning product in tablet and liquid forms
- Liquid soap for catheter washing
- Gloves for handling the chlorine-based cleaning products
- The soapy water and chlorine-based method: Plastic containers of varying shapes appropriate for male and female length catheters e.g. square or rectangular plastic containers or jugs with fitted lids, Milton Solo travel container, collapsible bowels for cleaning away from home
- Luminal flush: catheter tip and balloon syringes to flush catheter with SH, narrow nylon cleaning brush to insert through the lumen.

**Drying**

- Dry wipes
- Toothbrush holder for hanging catheters to drip dry
- Syringe for air flush

**Lubrication**

- Selection of lubricants (tubes, sachets)
- Paper towels for applying lubricant along the shaft
- Zip lock plastic bags for containing smaller quantities of lubricant when away from home

**Storage**

- Men: Digestive biscuit container to fit small pocket
- Women: slimline pencil case
- Both: opaque, nylon tubes with stoppers, of varying lengths and diameters to allow single or multiple catheters to be stored / carried away from home

**FINAL KIT FOR CATHETER REUSE IN THE MULTICATH TRIAL**

**Total number of components at completion of user testing = 5 items (excluding catheters)**


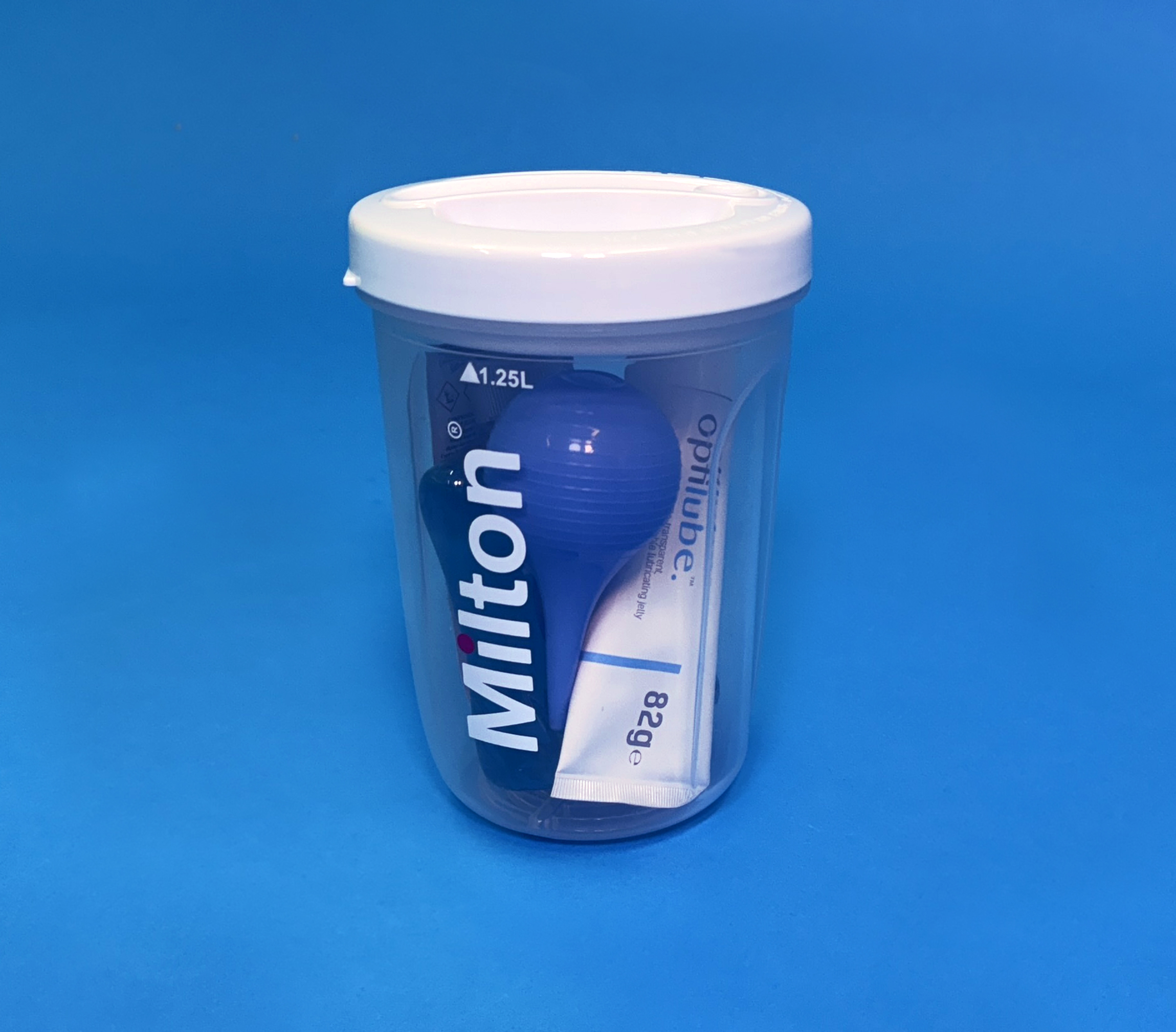

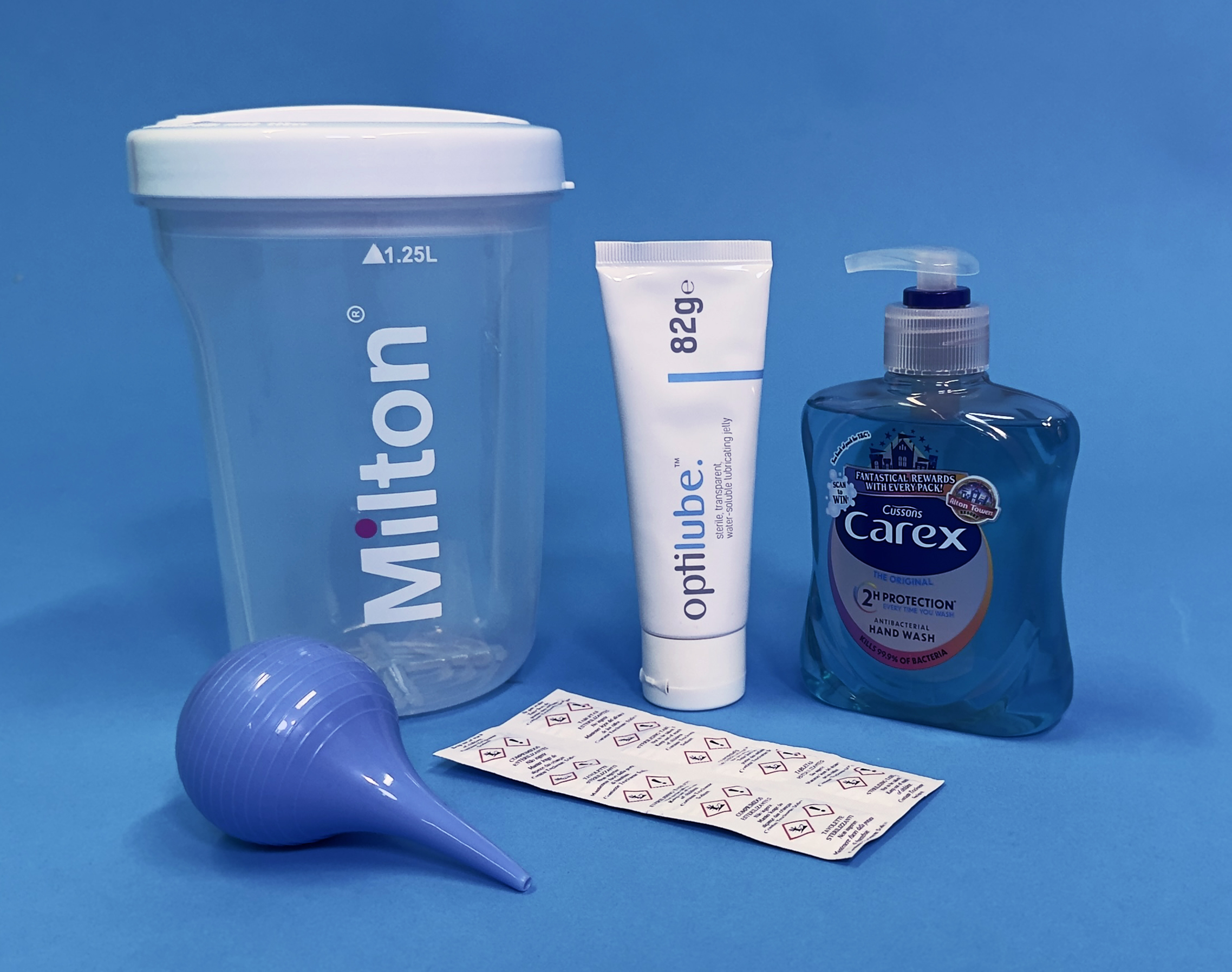

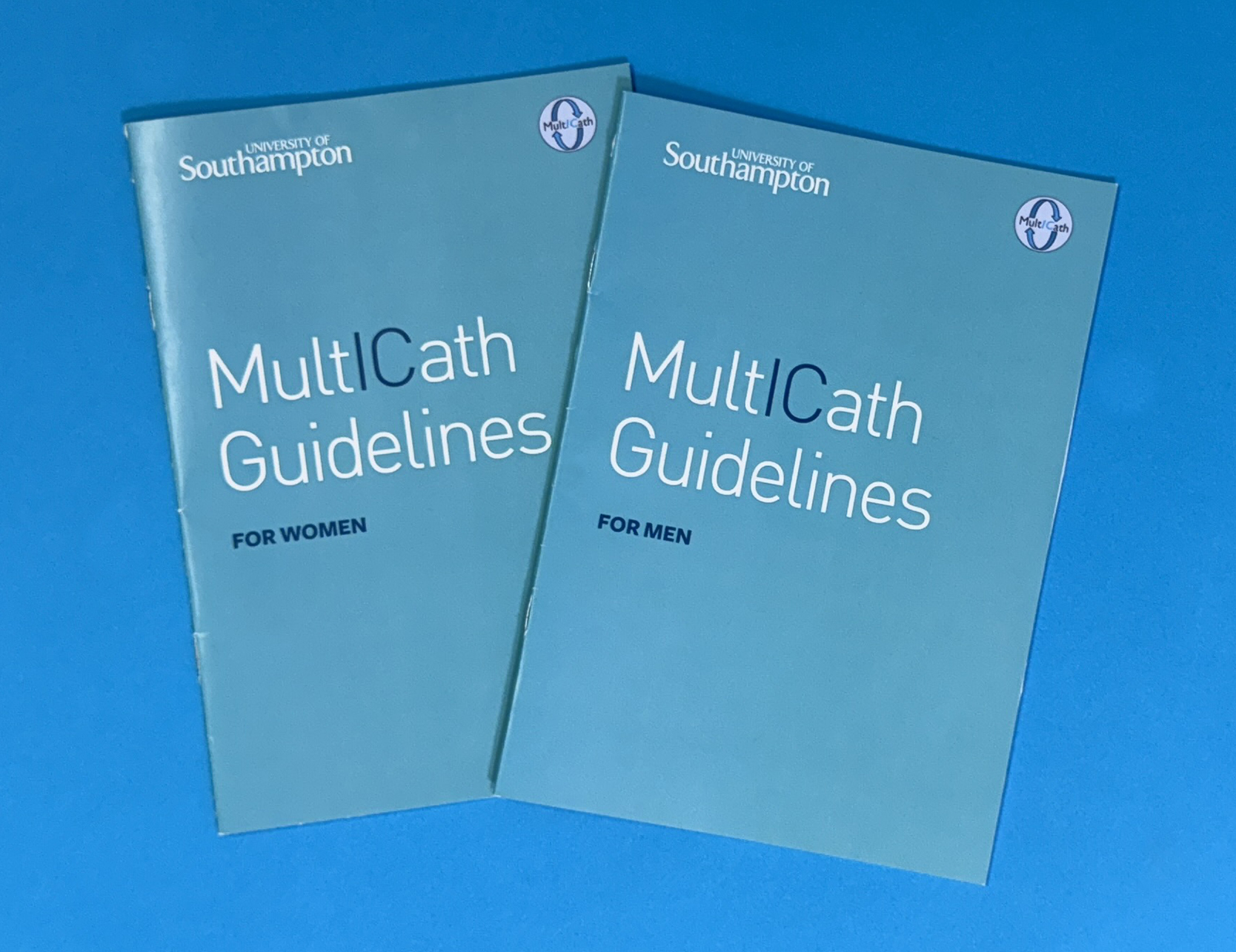

Supplement: Supplementary file 1 — Supporting Information 1: Microbiological analysis of urine Supporting Information 2: Microbiological analysis of plastic‐based catheters Supporting Information 3: Episcopic differential interference contrast (EDIC) analysis Supporting Information 4: Microbiological analysis of silicone catheters Supporting Information 5: Test method for cleaning catheters using soap and water and chlorine‐based cleaning solution (SW‐Cl) Supporting Information 6: Components reviewed for catheter cleaning and associated procedures necessary for catheter reuse. [file BCO2-6-e487-s002.docx]
