# Supplementary material for: How to clean a catheter: Development of an intervention for intermittent catheter reuse
Source: BJUI Compass. 2025 Feb 4;6(2):e487. doi: 10.1002/bco2.487 (PMC11794242; doi:10.1002/bco2.487)
Supplement: Supplementary file 2 — Supporting Information 7: Summary of user feedback, example quotes and modification to cleaning method and other processes for catheter reuse (separate file) [file BCO2-6-e487-s001.docx]

| **SUMMARY OF USER FEEDBACK, EXAMPLE QUOTES AND MODIFICATION TO CLEANING METHOD AND OTHER PROCESSES FOR CATHETER REUSE** | |
| --- | --- |
| **CLEANING** | |
| **User feedback** | **Amendment/modification to catheter cleaning process** |
| Users generally experienced few problems with the cleaning method and equipment but emphasised the need for the method to be adaptable for when away from home.  *“Using the reusable catheters at home hasn’t been a problem, …. the equipment that we were given for cleaning and sterilising was quite discreet.” (woman)*  *“Cleaning the catheters at home was very easy to do because obviously you’ve got your own facilities …. Away was a little bit more difficult because you are not always knowing what facilities you were going to have in hotel rooms or public bathrooms, that type of thing so there were a few problems while I was away from home.” (woman)* | Different size containers were provided for home and away and for male and female length catheters. |
| Users were concerned about the cleaning of the inside of the catheter lumen. | A luminal flush with a syringe was incorporated into the cleaning method. |
| The cleaning kit can be obvious if space is limited.  *“…. keeping the steriliser and all the bits and pieces out of sight particularly when we have visitors staying for the weekend. I did fine that quite difficult.” (woman)* | Items needed for the re-use intervention were kept to a minimum and provided in a discreet kit box. |
| Different Milton formulations were preferred for home or when out e.g. liquid Milton was more acceptable at home, but Milton tablets were lighter for travelling. | Milton was provided in both liquid and tablet form. For the Multicath trial, a bespoke tablet size was developed. |
| Odour from Milton and potential damage to hand skin or urothelium from repeated exposure to Milton was a concern. | Instructions for use (IFU) included advice on safety aspects of Milton including that sodium hypochlorite evaporates for catheter surfaces when dry. |
| Finding reliable hand washing facilities when away from home was important and could be difficult; familiar facilities e.g. at work were preferred | No action taken. Evidence that users need single-use catheters as well as reusable ones. |
| Users wanted multi-media instructions rather than only written materials. . | Participants were provided with a video (DVD and online), unisex booklets and a laminated card giving details of each process in easy-to-use steps. |
| **DRYING** | |
| Users found the catheters hard to dry completely.  *“The catheters seemed to take a long time to dry but I used to hang them on the little hanging thing *catheter holder) and then reuse them wet” (woman)*  *“…drying of catheters has been quite straight forward, we were given a number of different methods that we could use and depending on whether I was at home or away as to what method I used but it seemed to be quite straight forward. “(woman)* | Different drying methods were offered for testing e.g. paper towels, hanging from a toothbrush holder, a luminal air flush. |
| **LUBRICATION** | |
| Men found lubrication of the catheter shaft important. | A selection of lubricants was provided, and participants used their preferred type. |
| Women did not always need a lubricant.  *“When I was using the reusable catheters in the study I did lubricate, I tried lubricating them with the gel, but I actually found I preferred just to lubricate them under running water and then use them” (woman)* | Options for lubrication included using the catheter straight from the Milton or after rinsing under the tap. |
| Different methods of lubrication were needed for use at home and when out and preparation was found to be key.  *“I prepared myself prior to going away by lubricating six or seven of the catheters and take them with me in a clean bowl” (man)* | Different lubrication methods were provided including application with cleaned fingers or a paper towel (easier at home with reliable bathroom facilities) and a Ziplock bag containing lubricant (easier when out). |
| **STORAGE & PORTABILITY** | |
| Generally, when at home storage was not a problem unless sharing bathroom facilities or where co-habitants are not aware of the catheterisation.  *“I was fortunate…I was able to use a cupboard in the bathroom and the research project had kindly provided a holder for the catheters which I was able to put on the inside of the cupboard door so in actual fact the rest of the kit was on the bathroom shelf, but it really didn’t cause any problem” (man)* | A slimline ‘kit box’ was provided for discreet storage. |
| Users wanted discreet and easy to carry methods which look non-medical when taking catheters out.  *“Transporting the reusable catheters, I used the long narrow containers or rounded containers…I found that very successful, the larger one for transporting the used catheters and the narrower ones for putting clean catheters in to take with me” (man)*  *“While I was travelling, I took the Tupperware container we were given which was oblong shaped and if I was only away for a couple of days, I was able to put everything in there…… then I was able to the sterilise the catheters overnight.” (woman)*  They made suggestions about the type of storage that would suit their needs:  *“…. if you were playing snooker, your cue box kind of thing or something that like for men would be kind of discreet” (man)****hey had ideas about*** | Different options for catheter storage were provided. |
| The benefits of needing to take fewer catheters when travelling was noted.  *“On a day-to-day basis it is potentially less stuff to carry around than when using single use catheters” (woman)* | No action taken. |
| Men generally preferred to store their catheter in a pocket and needed a discreet container for this purpose. | A small round container was provided as well as opaque tubes with stoppers in male-catheter lengths and different widths for carrying one or multiple catheters. |
| Women needed a discreet container in which to store their catheter in their handbag. | A slimline pencil case was provided as well as opaque tubes with stoppers in female-catheter lengths and different widths for carrying one or multiple catheters. |
| Users wanted items to be kept to a minimum and double up, when possible, e.g. one container for the soapy water wash and then the Milton soak. | Small, discreet containers were provided in varied dimensions to suit male and female-length catheters. |
| ‘Smart’ packing was thought to be important for travelling, e.g. filling the Milton container with kit or other travel items. | The IFU included guidance on packing the catheter re-use kit in the Milton container for travelling. |
| **OVERALL EXPERIENCE OF CATHETER REUSE** | |
| Participants acknowledged the burden of reuse: *“When I first started the trial, I did find it a little bit of a nuisance sterilising the catheters but towards the end it became so much easier it just became part of regular life really” (woman)*  ….and that practice was needed to get used to new and additional processes and kit: *“… once I had the right gel for me and the right catheter for me the process became so automatic sometimes, I forgot whether I’d actually used a catheter as one forgets whether one has spent a penny. No problem at all.” (man)*. | The gradual introduction of more reuse activity over rounds of testing allowed for this adjustment. |
| Participants found pros and cons to both single-use and reuse of catheters and having the option of both was optimal: *“Being able to use the option of using both the reusable and the single use just was peace of mind that I could use whichever one was most convenient depending where I was.” (woman).* | The requirement to use either single-use or reusable catheters according to need (mix and match) was accommodated within all testing rounds. |
